# The Prevalence of Malnutrition in Iranian Elderly: A Review Article

**Published:** 2017-12

**Authors:** Hassan ABOLGHASEM GORJI, Mahtab ALIKHANI, Mohammad MOHSENI, Mohammad MORADI-JOO, Hajar ZIAIIFAR, Ahmad MOOSAVI

**Affiliations:** 1. Dept. of Health Services Management, School of Health Management and Information Sciences, Iran University of Medical Sciences, Tehran, Iran; 2. Health Management and Economics Research Center, Iran University of Medical Sciences, Tehran, Iran; 3. Iran Health Insurance Organization, Tehran, Iran; 4. Cancer Research Center, Shahid Beheshti University of Medical Sciences, Tehran, Iran; 5. Dept. of Health Management and Economics, School of Public Health, Tehran University of Medical Sciences, Tehran, Iran; 6. Dept. of Health and Community Medicine, Dezful University of Medical Sciences, Dezful, Iran

**Keywords:** Prevalence, Elderly, Malnutrition, Meta-analysis, Iran

## Abstract

**Background:**

The elderly population following the improvement in health status and life expectancy in developing countries is increasing. Malnutrition causes decreased quality of life and increased mortality in elderly. This study aimed to review systematically and meta-analysis of studies assessing the prevalence of malnutrition among Iranian elderly people over 60 yr of age using Mini Nutritional Assessment (MNA).

**Methods:**

This systematic review and meta-analysis was conducted in 2016 to estimate the overall malnutrition prevalence. Data were collected using the following keywords: prevalence, elderly, aging, malnutrition, nutrition, nutritional assessment, nutritional status, health status, mini nutritional assessment, MNA and Iran in PubMed, Scopus, Google Scholar, Iranmedex, Magiran, and SID. Computer software CMA: Two were applied to estimate the overall prevalence of malnutrition.

**Results:**

Seventeen of 811 articles were included in our analyses. The overall estimated prevalence of malnutrition among elderly based on the random effect model was 12.2% (95% CI 8–18.5). In subgroups, the prevalence of malnutrition among elderly living in home based on the fixed effect model was 9.2% (95% CI 7.1–11.9) and prevalence of malnutrition among elderly residents of nursing homes based on the random effect model was 21.6% (95% CI 12–38.6).

**Conclusion:**

Given the increase in the elderly population in future and the prevalence rate of malnutrition among them as well as the higher prevalence of malnutrition in elderly care centers, more attention to this population group is a matter of necessity.

## Introduction

Given the improvement in health status and medical services and accordingly increased life expectancy, the elderly population is increasing (1). The population of elderly aged 60 yr and over in developing countries is projected to reach 840 million by 2025 (2). According to demographic estimates, Iranian elderly population will constitute 14.7 percent of the country population at the end of the 20-Year Vision and will reach more than 26 million people in 2050 and the proportion of them to the total population will be about 23% (3, 4). Effects of aging cause significant changes in health and the performance of body system including the gastrointestinal system. These changes include decreased salivation, difficulty in swallowing, and delay in emptying of the stomach and esophagus as well as lower gastrointestinal movement (5) which all of these issues affect nutrition as one of the most important parts of health maintenance (6), and as a result elderly are a potentially vulnerable group at the risk of malnutrition (2). Drug use, loneliness, depression, lack of oral health, low quality of life, incidence of chronic diseases and frequent hospitalization influence elderly health and put them at higher risk of malnutrition and threat resulting from it (7). The undesirable nutritional status in addition to increased hospitalization also causes lower quality of life, increased length of stay in hospital and increased mortality among elderly (8–11). In addition it makes a favorable situation for the incidence of diseases such as diabetes, osteoporosis, cardiovascular disease and high blood pressure which these factors, in turn, cause the occurrence of other issues such as self-medication and side effects of medicines (12) and also create many health and socio-economic problems in the society (13). Mini Nutritional Assessment (MNA) is one of comprehensive and valid tools developed for assessing and determining the nutritional status among elderly which is used in the most of studies (14, 15). The MNA consists of an anthropometric assessment, a brief questionnaire about diet characteristics, global health and environment as well as a self-evaluation of health and nutritional status. The final score categorizes nutritional state as ‘well nourished’ (scores higher than 23.5), ‘at risk for undernutrition’ (scores from 17 to 23.5) and ‘undernourished’ (scores lower than 17) (16). Most published studies indicate the MNA to have high sensitivity and specificity and good predictive value for higher mortality, hospital admissions and other adverse outcomes (14, 16–18). Thus, the current study examined the literature to determine the prevalence of malnutrition among elderly over 60 yr of age in Iran with Mini Nutritional Assessment (MNA).

## Materials and Methods

### Search strategy

This systematic review and meta-analysis were conducted in 2016 using the approach described in “A Systematic Review to Support Evidence-Based Medicine (19)”. First, in order to identify all relevant publications on the prevalence of malnutrition among elderly over 60 yr of age in Iran a literature search was performed. Data were collected using the following keywords: prevalence, elderly, aging, malnutrition, nutrition, nutritional assessment, nutritional status, health status, mini nutritional assessment, MNA, and Iran. The following databases were searched: PubMed, Scopus, Google Scholar, Iran Medex, Magiran, and SID. There were no publication time limitations for this search. Potential articles were manually reviewed for relevance, and their reference lists were hand-searched to identify additional articles. Finally, we also consulted with experts and searched the gray literature. Reference management software (Endnote X5) was used to manage the references.

### Study Selection

Two reviewers (MM and AM) independently screened titles and abstracts for relevance. The full text of potentially relevant articles that evaluated malnutrition in elderly were obtained and assessed for eligibility. During this stage, in the case of conflicts between these two reviewers, consensus was reached using a third reviewer’s opinion. Studies were selected and included in the analyses based on the following inclusion criteria: original research, studies that focused on the prevalence of malnutrition in elderly over 60 yr of age, published in English or Persian and conducted in Iran. Conference presentations, case reports, and interventional and qualitative studies were excluded from the analysis. Subsequently, articles independently evaluated on the basis of the ‘Strengthening the Reporting of Observational Studies in Epidemiology’ (STROBE) checklist (20, 21).

### Data extraction

Two reviewers extracted the data using a standard data collection form (Table 1). The following information was extracted from each eligible study: Authors, year of publication, city, sample, sample size, prevalence of elderly with good nutrition, elderly at risk of malnutrition, and elderly with malnutrition (%).

**Table 1:** Characteristics of the studies

***Author, year of publication***	***City***	***Sample***	***Sample size***	***Good nutrition (%)***	***At risk of malnutrition (%)***	***Malnutrition (%)***
Tanjani PT et al: 2015 (25)	Iran	Elderly	1350	53.2	41.5	5.5
Nabavi, SH et al: 2015 (3)	Bojnourd	All the elderly	120	30	62.2	7.5
Lashkarboloki, F et al: 2015 (26)	Gorgan	Elderly	541	50.5	44.7	4.8
Davvalo Khongar, P et al: 2015 (27)	Tehran	Elderly residents of nursing homes	245	35.1	55.9	9
Nazemi, L et al: 2015 (11)	Tehran	Elderly residents of nursing homes	263	20.9	68.8	10.3
Abdolhasan Naghibi, S et al: 2014 (28)	Sari	Elderly residents of nursing homes	104	25	40.38	38.62
Vafaei, Z et al: 2013 (29)	Isfahan	Rural elderly	370	63.5	32.7	3.8
Payahoo, L et al: 2013 (30)	Tabriz	Free-living elderly	184	47.3	46.7	6
Ebrahimi Fakhar, MR & Zand, S: 2012 (31)	Arak	Elderly residents in nursing homes	199	27.1	53.3	19.6
Masomy, N et al: 2012 (32)	Rasht	Retired senile	194	87.1	12.9	4
Mokhber, et al: 2011 (33)	Razavi Khorasan	Free-living elderly people	1565	46.1	43.3	10.6
Pasdar, Y et al: 2011 (34)	Kermanshah	Elderly residents of nursing homes	140	46.4		53.6
Saeidlou, SN et al: 2011 (35)	Urmia	Elderly residents of nursing homes	106	12.26	38.68	49.06
Amirkalali, B et al: 2010 (16)	Tehran	Kahrizak charity foundatio	221	53.4	43.4	3.2
Afkhami A et al: 2008 (36)	Tehran and Shemiranat	Elderly residents of nursing homes	290		56.2	12.8
Aliabadi, M et al: 2007 (37)	Khorasan Razavi	Free-living elderly	2000	42.7	45.3	12
Eshaghi, SR et al: 2007 (24)	Isfahan	Elderly	248		37	3

### Data analysis

The computer software CMA: 2 (Comprehensive Meta-Analysis) (Englewood, NJ, USA) applied to perform the meta-analysis and estimated the overall malnutrition prevalence. The presence of heterogeneity across the studies was assessed by the I^2^ statistic (22). Statistical significance for publication bias was based on a *P*-value of <0.05. The point prevalence 95% (95% confidence interval [CI]) was displayed using the forest plot so that the size of each square represents the sample size and the lines on each side of the square indicate the confidence interval. Microsoft Office Excel 2010 was used to draw graphs. Funnel plot was applied to evaluate the possibility of publication bias (23).Three forest plots were drawn for analysis.

First, analysis of all articles was conducted. Then two sub-groups were formed: one composed of articles related to the elderly living at home, and the other of the elderly residents of nursing homes.

## Results

**Fig. 1:**
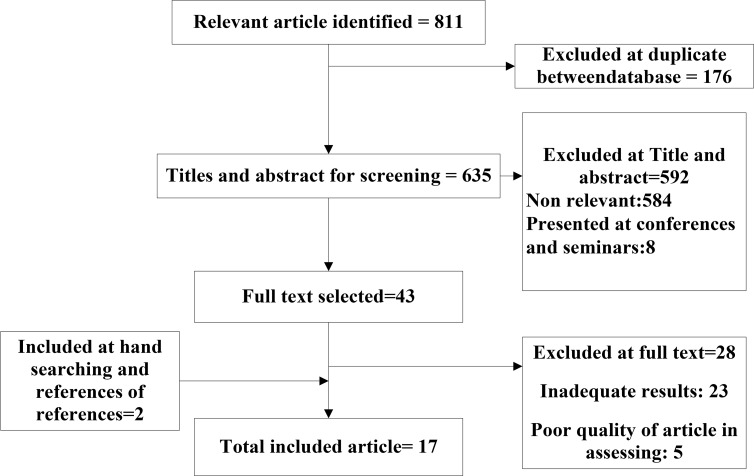
Flow diagram of literature search

**Fig. 2:**
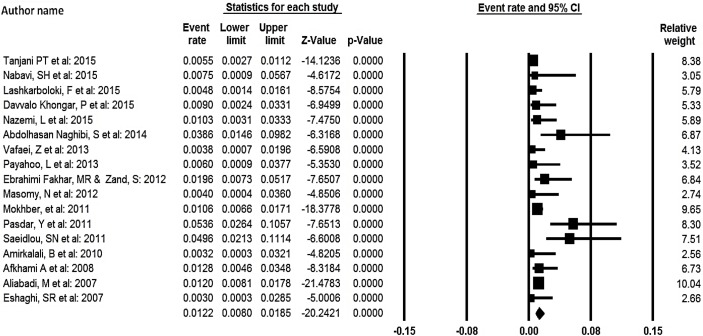
Prevalence of malnutrition in elderly over 60 yr old

Of 811 studies identified in the initial search, 17 studies with 8140 persons were included in the final analysis. These studies were conducted between 2007 and 2015. Of 17 studies, seven studies were related to the prevalence of malnutrition among elderly residing in elderly home. While the lowest percentage of prevalence of malnutrition among elderly was associated with the study conducted (3%) in Isfahan (24), the highest percentage was related to the study conducted on elderly in Tehran residing in elderly home (11). Fig. 1 shows the process of the selection of studies. Table 1 also shows the characteristics of the mentioned studies. In the meta-analysis of 17 studies consisting of 8140 elderly over 60 yr old, the overall prevalence of malnutrition based on the random effect model was 12.2% (95% CI 8–18.5) (Fig. 2). Overall, 95% CI for the prevalence was drawn for each study in the horizontal line format (Q=46.7 df =16, *P*<0.001, I^2^= 65.7).

**Fig. 3:**
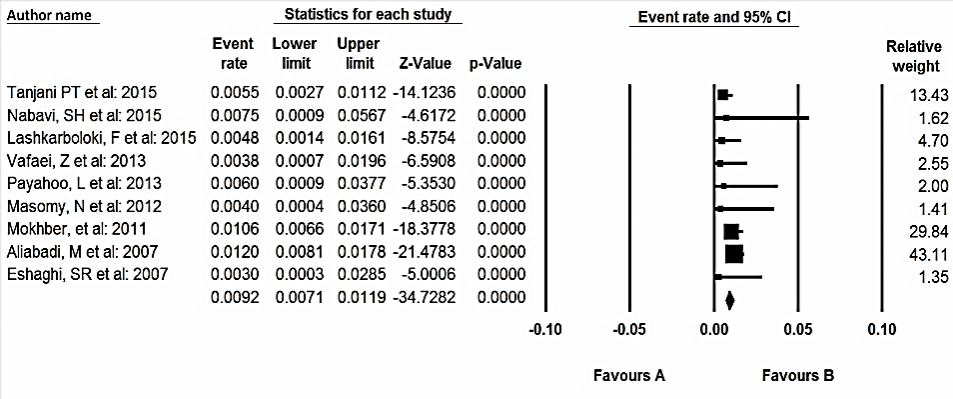
Prevalence of malnutrition among elderly over 60 yr old living in home

**Fig. 4:**
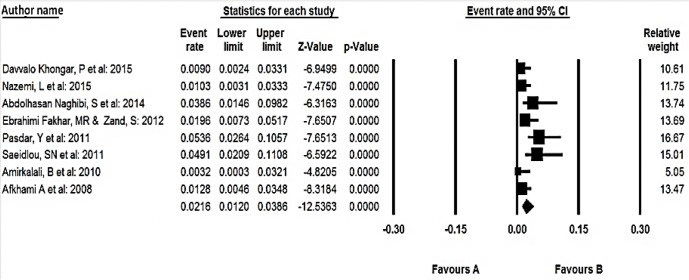
Prevalence of malnutrition among elderly over 60 yr old residents of nursing homes

For the meta-analysis of the prevalence of malnutrition among elderly living in home (Fig. 3) and elderly residents of nursing homes (Fig. 4), we performed subgroup analysis for studies.

In the meta-analysis of nine studies consisting of 6572 elderly over 60 yr old, the overall prevalence of malnutrition based on the fixed effect model was 9.2% (95% CI 7.1–11.9). 95% CI for the prevalence was drawn for each study in the horizontal line format (Q=7.92 df=8, *P*=0.43, I^2^=0).

In the meta-analysis of eight studies consisting of 1568 elderly over 60 yr old, the overall prevalence of malnutrition based on the random effect model was 21.6% (95% CI 12–38.6). Of 95% CI for the prevalence was drawn for each study in the horizontal line format (Q =17.2 df=7, *P*<0.001 I^2^=59.1).

**Fig. 5:**
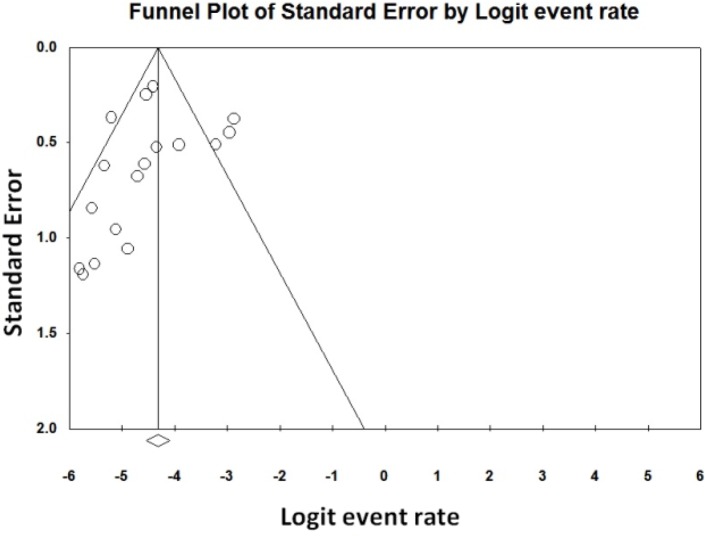
Funnel plot

To evaluate the publication bias, funnel plot was applied (Fig. 5). The result of this funnel plot show there was possibility publication bias among studies. Publication bias could affect the results of our meta-analysis.

## Discussion

The overall prevalence of malnutrition among elderly, among elderly living in home and among elderly residents of nursing homes was 12.2%, 9.2%, and 21.6%, respectively. The rate of malnutrition among elderly in studies using MNA was reported to be between 3% and 10% and the risk of suffering from malnutrition were 20% to 50% (38–42).

Moreover, in a study, based on MNA tool, 19.9% of elderly residing in the society and non-hospitalized suffered from malnutrition, 58% of them were at risk of malnutrition and 22.1% had desirable nutritional status (43). The prevalence of malnutrition among elderly residing in the society in a study was also reported to be 5% to 10% (44). The estimation of the prevalence of malnutrition among elderly residing in elderly homes in Iran was 21.6%, which is higher than the rate for those elderly living freely in the society. About 15% to 71% of elderly population in nursing homes suffered from malnutrition and about 40% to 60% of them were at risk of malnutrition (45–47). Moreover, the prevalence of malnutrition among elderly in western societies in elderly homes and hospital in a study was reported to be 37% (38). Being far from family can provide ground for physical and mental diseases, so that married people compared to divorced people, and also those elderly living with their families compared to those living alone visited physicians and/or were hospitalized less; this finding indicates the positive impact of family members relationship on quality of life (48). Elderly residing in elderly homes compared to those living in their own homes are more vulnerable in terms of nutritional disorders (46, 49, 50). By entering elderhood the rate of disabilities is gradually increasing, therefore, the prevalence of movement restrictions at 76 yr of age and above reach even to 50% which these restrictions, in turn, cause the dependency of elderly (31) and them with increasing movement disabilities, the likelihood of transferring elderly to elderly homes and care centers increases (51). Since the social isolation followed by the loss of appetite causes exacerbation of the reduction of food intake and increased the likelihood of the risk of malnutrition (52) thus care goals of health authorities should be more focused on physical health needs of elderly residing in elderly homes. Furthermore, problems of those elderly separated from their families can be informed and the culture of caring elderly in warm family environment can be strengthened through public education at the society level (31).

In order to reduce malnutrition among elderly two types of activity are needed. First, regarding those elderly suffering from malnutrition which preventive and curative programs should be conducted for them. Second, for those elderly at the risk of malnutrition; given the effects of aging on physical status and the likelihood of occurrence of malnutrition in future among elderly at risk of malnutrition, appropriate preventive and supportive programs for this group who constitutes a high percentage should also be conducted.

## Conclusion

The prevalence of malnutrition among Iranian elderly over 60 yr of age was 12.2% and among elderly residing in elderly homes was 21.6%. Two points should be taken into account; first, the increase in the number of elderly in the future and the necessity of attention to their status and second, the higher prevalence of malnutrition among those elderly residing in elderly homes as well as the importance of caring elderly in warm family environment.

## Ethical considerations

Ethical issues (Including plagiarism, informed consent, misconduct, data fabrication and/or falsification, double publication and/or submission, redundancy, etc.) have been completely observed by the authors.
